# Joint Monitoring and Early Warning of SARS-CoV-2 in Outdoor PM_2.5_ and Wastewater

**DOI:** 10.3390/v18070788

**Published:** 2026-07-19

**Authors:** Wenli Wang, Haizhou Liu, Dan Wang, Zhiwei Chen, Yu Jiang, Jianjun Xiang, Jing Li, Xiaoyang Zhang, Chuancheng Wu

**Affiliations:** 1 School of Public Health, Fujian Medical University, Fuzhou 350122, China; wangwenli@fjmu.edu.cn (W.W.); wangdan1376@163.com (D.W.); jiangyu@fjmu.edu.cn (Y.J.); jianjun.xiang@fjmu.edu.cn (J.X.); leejing@fjmu.edu.cn (J.L.); 2 Key Laboratory of Environment and Health, Fujian Province University, Fuzhou 350122, China; 3 Department of Infectious Diseases, Fuzong Clinical Medical College, Fujian Medical University, Fuzhou 350122, China; fzzyygrkliu@fjmu.edu.cn; 4 Department of Infectious Diseases, 900th Hospital of PIA Joint Logistics Support Force, Fuzhou 350004, China; 5 The Affiliated Fuzhou Center for Disease Control and Prevention, Fujian Medical University, Fuzhou 350200, China; chenzhiwei@fjmu.edu.cn

**Keywords:** PM_2.5_, wastewater, SARS-CoV-2, COVID-19, joint monitoring, early warning, lag effect

## Abstract

Background: Fine particulate matter (PM_2.5_) and wastewater may carry severe acute respiratory syndrome coronavirus 2 (SARS-CoV-2) and provide early signals for coronavirus disease 2019 (COVID-19) surveillance. This study investigated environmental factors affecting SARS-CoV-2 distribution in PM_2.5_ and wastewater in Fuzhou. Methods: PM_2.5_ and wastewater samples collected from January to December 2023 were tested for SARS-CoV-2 RNA using reverse transcription quantitative polymerase chain reaction (RT-qPCR). Associations between air pollutants and meteorological factors were analyzed using generalized additive models (GAMs) and distributed lag nonlinear models (DLNMs). Results: SARS-CoV-2 was detected in 45.9% of PM_2.5_ samples. PM_2.5_ viral concentrations were positively associated with CO, PM_2.5_, and atmospheric pressure, and negatively associated with temperature and sunshine duration. Wastewater viral concentrations were positively associated with relative humidity and precipitation and negatively associated with NO_2_ and SO_2_. PM_2.5_ signals showed the strongest lag effect at approximately 2 days, while wastewater signals peaked at lag 0. The lag effect herein describes the time interval between environmental SARS-CoV-2 signals and predicted COVID-19 outpatient cases. Elderly populations showed higher environmental sensitivity, with sex-specific differences observed. Conclusions: SARS-CoV-2 signals in PM_2.5_ and wastewater reflected COVID-19 trends. Integrated multi-media monitoring may improve early warning and support targeted public health interventions.

## 1. Introduction

Emerging infectious diseases, such as Coronavirus Disease 2019 (COVID-19), have highlighted the urgent need to establish effective pathogen environmental monitoring and early warning systems in urban settings [1,2]. Although clinical case reporting remains the cornerstone of epidemic response, environmental matrices such as atmospheric particulate matter (PM_2.5_) and urban wastewater have increasingly emerged in recent years as complementary or alternative approaches for early warning, particularly offering unique advantages for monitoring asymptomatic infections and assessing population-level viral load dynamics [3,4].

Severe Acute Respiratory Syndrome Coronavirus 2 (SARS-CoV-2) has been detected in both respiratory aerosols and wastewater, suggesting its potential for widespread persistence and transmission in the external environment [5,6]. Previous studies have demonstrated that wastewater-based epidemiology (WBE) can serve as an effective tool for early warning of COVID-19 outbreaks [7,8], and some evidence has also indicated a positive correlation between PM_2.5_ concentrations and virus transmission rates or case fatality rates [9,10,11]. However, systematic studies that integrate both atmospheric and wastewater matrices for viral monitoring and prediction remain limited.

This study used reverse transcription quantitative polymerase chain reaction (RT-qPCR) to detect SARS-CoV-2 RNA in outdoor PM_2.5_ and wastewater samples collected in Fuzhou. By integrating air quality and meteorological data, the temporal distribution of the virus in the environment and its associations with environmental factors were systematically assessed. Furthermore, the lagged effects of viral presence on COVID-19 case trends were examined, providing a scientific basis for establishing a multi-media virus joint monitoring and early warning system.

## 2. Materials and Methods

### 2.1. Sample Collection and Viral Detection Method

The PM_2.5_ samples were collected from three sampling sites located in the urban center, the urban–rural fringe, and the rural areas. Wastewater samples were collected from the A wastewater treatment plant in Fuzhou City. The sampling period was from 1 January to 31 December 2023, with a sampling frequency of once every three days. Viral RNA was extracted using the TIANMicrobe Magnetic Patho-DNA/RNA Kit (Fuzhou Jingrui Biotechnology Co., Ltd., Fuzhou, Fujian, China). RT-qPCR was performed on a QuantStudio 5 Real-Time PCR System (Applied Biosystems, Thermo Fisher Scientific, Waltham, MA, USA) using the Evo M-MLV One Step RT-qPCR Kit (Fuzhou Xianghua Bioengineering Co., Ltd., Fuzhou, Fujian, China) and SARS-CoV-2 amplification primers (Fuzhou Ruizhen Biotechnology Co., Ltd., Fuzhou, Fujian, China) to detect the viral load of SARS-CoV-2.

### 2.2. Data Sources

The air quality data were obtained from the China National Urban Air Quality Real-Time Publishing Platform, including PM_2.5_, PM_10_, NO_2_, 8 h average ozone concentration (O_3_-8 h), CO, and SO_2_. Meteorological data were obtained from the National Meteorological Science Data Center, including precipitation (mm), daily mean wind speed (m/s), mean atmospheric pressure (hPa), mean air temperature (°C), mean relative humidity (%), sunshine duration (h), and minimum visibility (m).

Hospital data were derived from outpatient records of two COVID-19 sentinel hospitals and 11 respiratory disease hospitals. According to the China Influenza Surveillance Protocol (2017 edition), these COVID-19 sentinel hospitals conducted SARS-CoV-2 testing primarily among outpatients presenting with influenza-like illness (ILI), rather than being limited to hospitalized or critically ill patients. All laboratory-confirmed COVID-19 case data were provided by the Fuzhou Center for Disease Control and Prevention. Diagnoses were classified according to the International Classification of Diseases, Tenth Revision (ICD-10) codes J00-J99, covering various respiratory diseases. The dataset contains no personally identifiable information.

The daily SARS-CoV-2 positivity rate at the sentinel hospitals was used as a reference indicator for COVID-19 incidence to estimate the daily potential COVID-19 incidence in Fuzhou. Meanwhile, the daily number of outpatient visits for respiratory diseases at the 11 monitored hospitals was used as a proxy for suspected cases. The estimated number of predicted COVID-19 cases was then calculated by multiplying the daily SARS-CoV-2 positivity rate by the total number of outpatient visits for respiratory diseases.

### 2.3. Statistical Analysis

Spearman correlation was used to assess associations. When the absolute value of the correlation coefficient (|ρ|) exceeded 0.8, multicollinearity was deemed present, and the corresponding variables were excluded from subsequent modeling. A Generalized Additive Model (GAM) was used to model nonlinear relationships. Considering that COVID-19 cases represent a low-probability event in the general population of Fuzhou, the daily case counts were modeled as a Poisson-distributed dependent variable. A log link function was used, and smoothing spline functions were introduced for meteorological and air quality variables to capture potential nonlinear effects. The model formula was as follows:

$$logE\left(Y_{w}\right)=b+\sum s(x_{i},df)$$

*Y_w_* represents the number of COVID-19 cases in week w; E(Y_w_) denotes the expected number of COVID-19 cases in week w; *b* is the intercept term of the model; s() indicates the smoothing spline function; *x_i_* represents the independent variables; and df refers to the degrees of freedom for the spline function of each variable.

Considering the incubation period of COVID-19, a distributed lag nonlinear model (DLNM) was applied to assess the lagged and nonlinear effects of environmental exposures on the number of COVID-19 cases, with a maximum lag of two weeks. Due to the limited sample size, cross-basis functions were constructed separately for each variable to analyze their individual lag effects. The model was expressed as follows:

$$Y_{t}\sim Poisson\left(u\right)=\alpha +cb\left(M,df,lag,df\right)+ns\left(Week,df\right)+\sum i(\beta_{i}X_{i,t})$$

In the model, t denotes the observation week; *Y_t_* represents the number of COVID-19 cases in week t; *α* is the intercept; cb denotes the cross-basis function, which is used to evaluate the nonlinear exposure–response relationships and lag effects between meteorological and air quality factors and COVID-19 cases; ns represents the natural spline function; week is included to control for temporal trends; *X_i, t_* indicates the value of the i-th covariate at time t; and i represents the regression coefficient of the i-th covariate.

## 3. Results

### 3.1. Basic Characteristics of Meteorological and Air Quality Factors

Descriptive statistics of meteorological and air quality factors from January to December 2023 are presented in Table 1. Overall, the air quality in Fuzhou City was generally good, and the concentrations of most pollutants met the National Ambient Air Quality Standards. However, according to the World Health Organization guideline values, the compliance rates of PM_10_, PM_2.5_, and O_3_-8 h were lower than those specified by the national standards. Most air pollutants peaked in winter and reached their lowest levels in summer, whereas O_3_-8 h followed an opposite seasonal pattern.

### 3.2. Distribution Characteristics and Influencing Factors of SARS-CoV-2 in PM_2.5_

**Figure 1:**
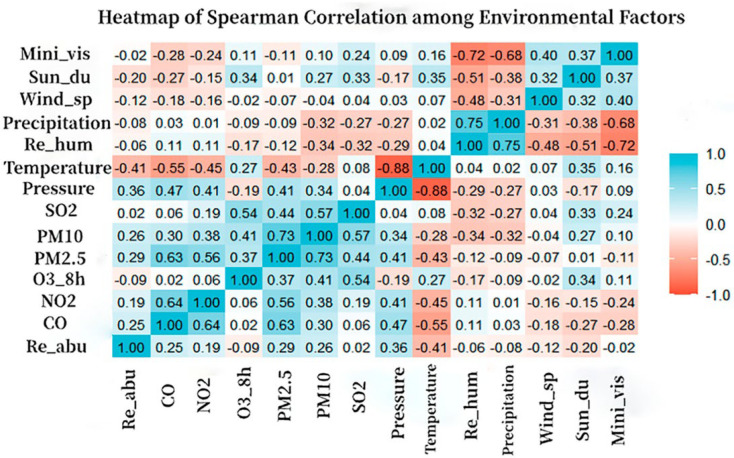
Correlation between SARS-CoV-2, air pollutants, and meteorological factors. Mini_vis: minimum visibility; Sun_du: sunshine duration; Wind_sp: wind speed; Re_hum: relative humidity; Re_abu: relative abundance of SARS-CoV-2 in PM_2.5_.

Among the 122 collected samples, 56 samples tested positive for SARS-CoV-2, with an overall detection rate of 45.9%. As shown in Figure 1, Spearman correlation analysis revealed that SARS-CoV-2 concentrations in PM_2.5_ samples were positively correlated with CO, PM_10_, PM_2.5_, NO_2_, and atmospheric pressure, while showing a significant negative correlation with temperature and sunshine duration.

Furthermore, multiple linear regression analysis (Table 2) confirmed that CO and atmospheric pressure were positive influencing factors, whereas temperature and sunshine duration were negative influencing factors.

### 3.3. Distribution Characteristics and Influencing Factors of SARS-CoV-2 in Wastewater

**Figure 2:**
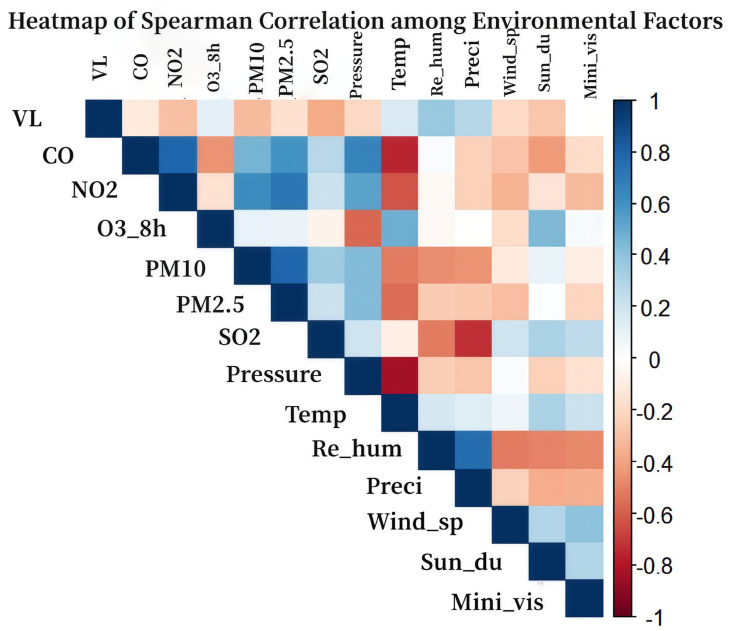
Correlation matrix of SARS-CoV-2 viral loads in wastewater with air quality and meteorological factors. VL: viral load; Temp: temperature; Re_hum: relative humidity; Preci: precipitation; Wind_sp: wind speed; Sun_du: sunshine duration; Mini_vis: minimum visibility.

As shown in Figure 2, Spearman correlation analysis indicated that SARS-CoV-2 concentrations in wastewater were positively correlated with relative humidity and precipitation, but negatively correlated with NO_2_, PM_10_, SO_2_, and sunshine duration. However, results from the multiple linear regression analysis (Table 3) showed that only SO_2_ was significantly associated with viral concentration.

**Figure 3:**
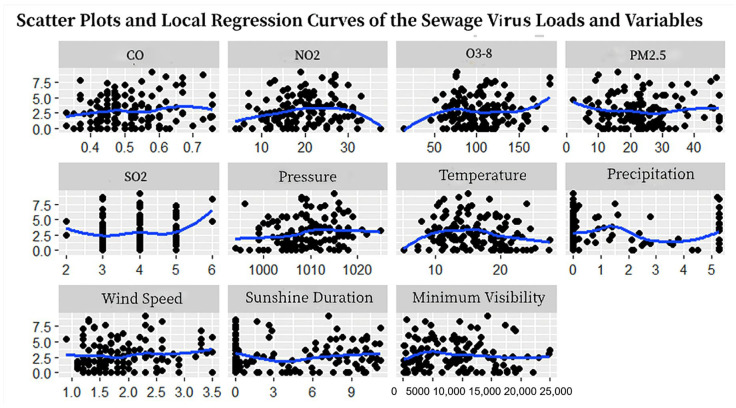
Scatter plots and locally weighted regression curves between environmental variables and the logarithmic values of SARS-CoV-2 load. Only variables showing significant nonlinear trends are presented. Linear associations are summarized in Table 2 and Table 3. The blue lines represent locally weighted regression smooth curves, illustrating the nonlinear trends between each environmental variable and the log-transformed viral load.

Further, locally weighted regression was applied to visualize the relationships between the log-transformed viral concentrations and each environmental factor (Figure 3). The results revealed nonlinear relationships between NO_2_, O_3_-8 h, SO_2_, and viral load, while other variables showed no obvious nonlinear trends.

**Figure 4:**
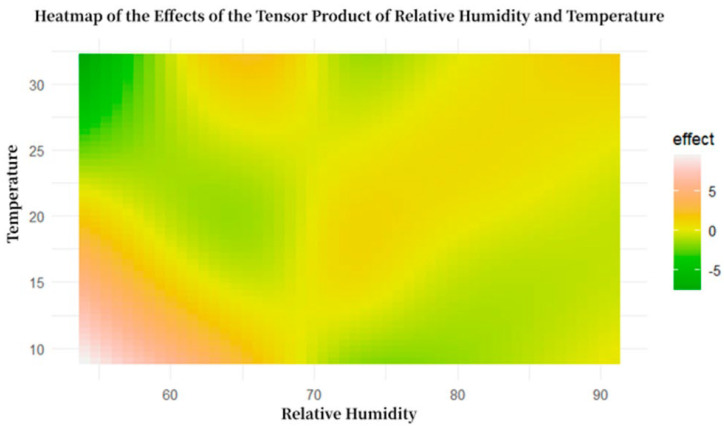
Heatmap of the effects of average relative humidity and mean temperature.

In addition, a GAM incorporating a tensor product interaction term ti (temperature, relative humidity) was constructed, where ti represents the tensor product interaction term for the two variables. The results (Figure 4) demonstrated that low-temperature and low-humidity conditions exerted an enhancing effect on viral concentration, whereas high-temperature and high-humidity conditions showed no significant impact on viral load.

### 3.4. Association Between Environmental Factors and COVID-19 Cases

In 2023, a total of 4405 suspected cases were screened at sentinel hospitals in Fuzhou, among which 457 cases were confirmed as COVID-19. Of these, 242 were male, and 215 were female, and the difference in detection rates between sexes was not statistically significant. Age-stratified (Table 4) analysis showed that the 22–49 years age group had the highest number of confirmed cases, while the ≥65-year age group had the lowest positivity rate, with a statistically significant difference among age groups.

**Figure 5:**
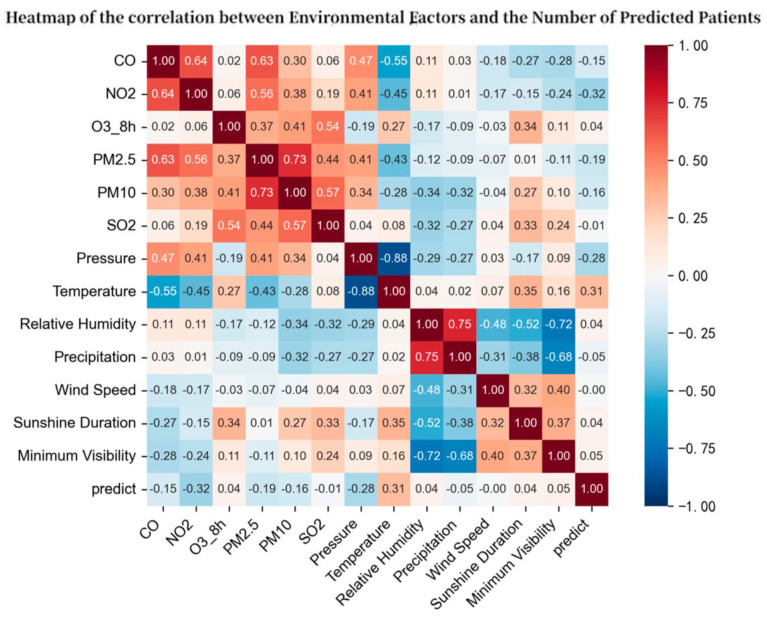
Correlation analysis between COVID-19 case numbers and meteorological and air quality factors in Fuzhou.

Spearman correlation analysis (Figure 5) indicated that the number of COVID-19 cases was negatively correlated with CO, SO_2_, and atmospheric pressure, but positively correlated with O_3_-8 h and temperature. Because temperature and pressure were highly correlated, they were not included simultaneously in subsequent modeling.

**Figure 6:**
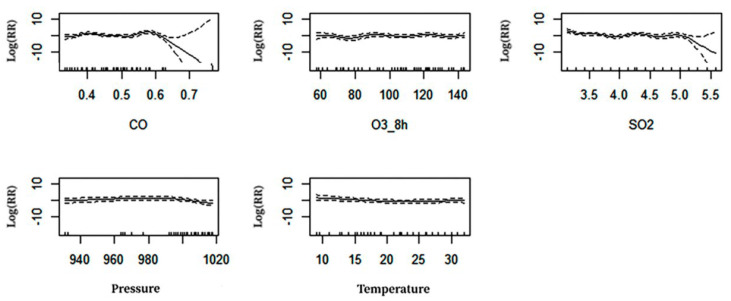
Exposure-response curves between air pollutants, meteorological factors, and COVID-19 incidence fitted by the generalized additive model. The dashed lines represent the 95% confidence intervals. An effect is considered statistically significant when the 95% confidence interval does not cross the null line (Log(RR) = 0, i.e., RR = 1).

Results from the GAM (Figure 6) showed that the exposure-response relationships between meteorological factors and COVID-19 incidence were generally not substantial, except that the risk decreased when CO and SO_2_ concentrations rose above certain levels. Other environmental factors did not exhibit clear exposure-response relationships with COVID-19 incidence. Formal significance tests for the GAM smooth terms were performed. Among all environmental factors, atmospheric pressure and temperature showed statistically significant associations with COVID-19 incidence (*p* < 0.05), while CO, O_3_-8 h, and SO_2_ did not reach statistical significance (*p* > 0.05).

**Figure 7:**
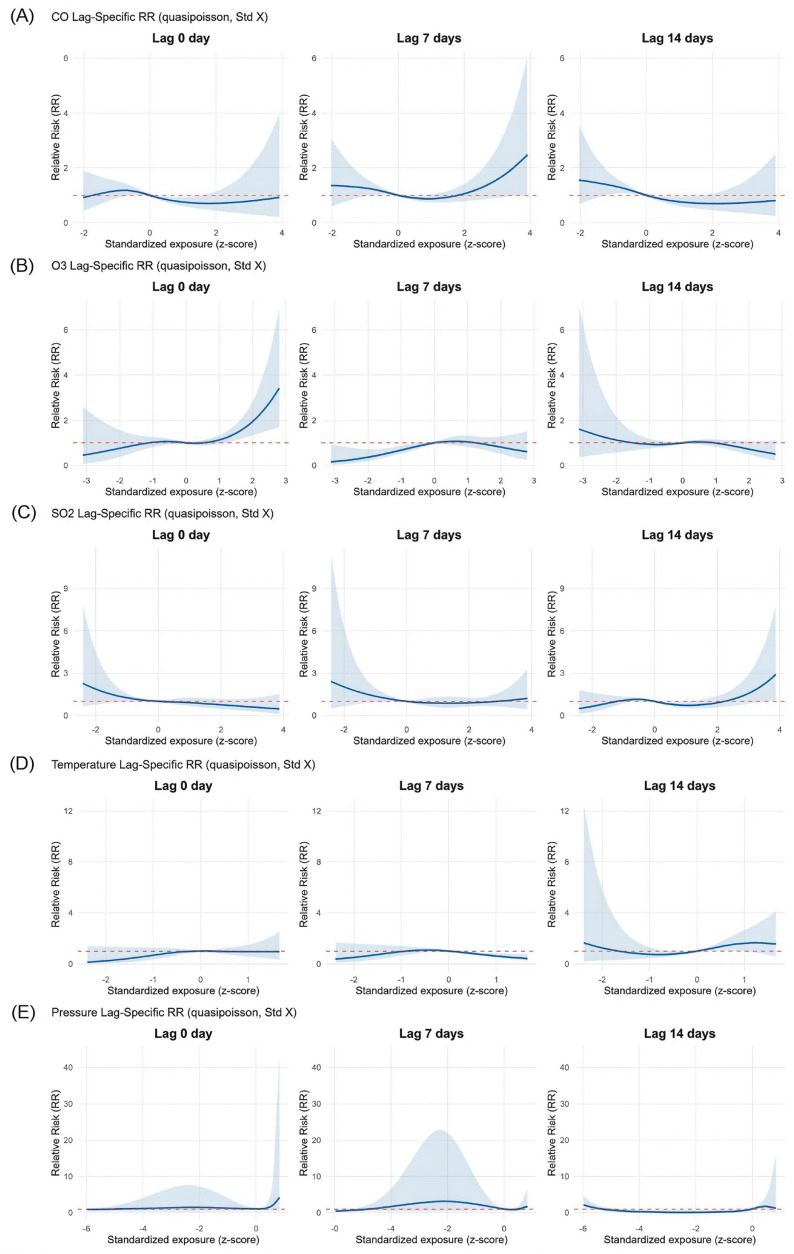
Distributed lag relative risks of different environmental factors on COVID-19 incidence at 7-day and 14-day lag periods. (**A**) CO; (**B**) O_3_-8 h; (**C**) SO_2_; (**D**) temperature; (**E**) atmospheric pressure. The red horizontal dashed line represents RR = 1 (the null line). An effect is considered statistically significant when the 95% confidence interval does not cross the null line.

The DLNM (Figure 7) showed that the relative risk associated with CO varied across different lag periods. After a lag of 7 days, the risk of COVID-19 incidence showed a decreasing then increasing trend with rising CO concentration. At lag 14 days, the relative risk exhibited a decreasing trend as CO concentration increased. The effect of O_3_-8 h was most pronounced at lag 0 days, with a sharp increase in risk at high concentrations, although the confidence intervals were wide and the estimates unstable. Low atmospheric pressure was associated with an increased risk of incidence, with the effect strongest at a lag of 7 days. Formal significance tests for the DLNM indicated that the overall effects of all environmental factors across the lag period were statistically significant (*p* < 0.05).

### 3.5. Lagged Effects and Population Heterogeneity of PM_2.5_-Carried SARS-CoV-2 on Predicted COVID-19 Cases

**Figure 8:**
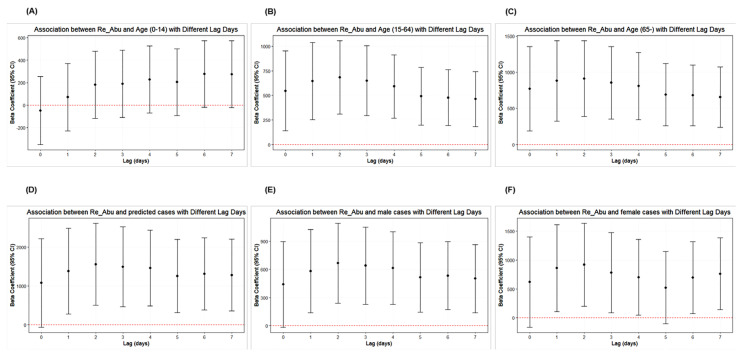
Lagged effects of PM_2.5_-bound SARS-CoV-2 on predicted COVID-19 cases across different age and gender groups. (**A**–**C**) Age groups: (**A**) 0–14 years, (**B**) 15–64 years, (**C**) ≥65 years; (**D**) All-age group; (**E**,**F**) Gender groups: (**E**) Males, (**F**) Females. “Re_abu” denotes the relative abundance of SARS-CoV-2 in PM_2.5_ samples. The red dashed line represents the null line (regression coefficient β = 0). An effect is considered statistically significant when the 95% confidence interval does not cross this line.

After adjusting for meteorological and air quality factors, a GLM was constructed to perform stratified analyses by age and sex. As shown in Figure 8, the viral load of SARS-CoV-2 carried by PM_2.5_ had a positive effect on COVID-19 cases across all age groups, with a 1–7 day lag, and the strongest effect was observed at a 2-day lag. Among different age groups, the ≥65 years old group showed the largest increase during lag days 0–7, with each unit increase in viral concentration associated with 385.11–1437.32 additional cases, while no significant effect was observed in the 0–14 years old group. Sex-stratified analysis showed that males exhibited a unimodal pattern with an increase followed by a decrease, whereas females displayed a bimodal pattern with an increase, a decrease, and a subsequent increase. Despite these differences, both males and females reached their peak effects at a 2-day lag, with each unit increase in viral concentration associated with a rise of 241.79–1092.12 cases in males and 197.12–1636.26 cases in females.

### 3.6. Lagged Effects and Population Heterogeneity of Wastewater SARS-CoV-2 on Predicted COVID-19 Cases

**Figure 9:**
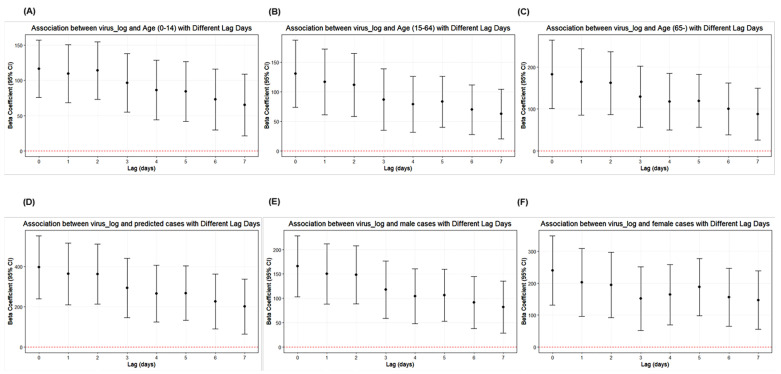
Lagged effects of log-transformed SARS-CoV-2 concentrations in wastewater on predicted COVID-19 cases across different age and gender groups. (**A**–**C**) Age groups: (**A**) 0–14 years, (**B**) 15–64 years, (**C**) ≥65 years; (**D**) All-age group; (**E**,**F**) Gender groups: (**E**) Males, (**F**) Females. “virus_log” denotes the log-transformed concentration of SARS-CoV-2 in wastewater samples. The red dashed line represents the null line (regression coefficient β = 0). An effect is considered statistically significant when the 95% confidence interval does not cross this line.

Age- and sex-stratified analyses based on the general linear model are presented in Figure 9. The log-transformed SARS-CoV-2 concentration in wastewater exhibited a positive lag effect on predicted COVID-19 cases across all age groups, with the strongest effect observed at lag 0 days. For the overall population, each one-unit increase in log viral concentration corresponded to an increase of 238.80–552.71 predicted cases, while in the ≥65-year group, the increase ranged from 100.90 to 264.66 cases. Sex-stratified analysis indicated that both males and females reached their peak lag effects at lag 0 days. For each one-unit increase in log viral concentration, the number of predicted cases increased by 103.05–228.50 in males and 131.64–349.01 in females.

### 3.7. Multi-Media Lagged Effects and Population Heterogeneity of PM_2.5_ and Wastewater SARS-CoV-2 on Predicted COVID-19 Cases

**Figure 10:**
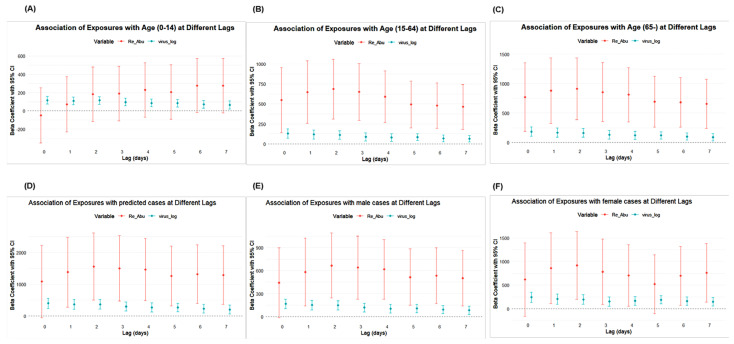
Lagged effects of SARS-CoV-2 in PM_2.5_ and wastewater on predicted COVID-19 cases across different age and gender groups. (**A**–**C**) Age groups: (**A**) 0–14 years, (**B**) 15–64 years, (**C**) ≥65 years; (**D**) All-age group; (**E**,**F**) Gender groups: (**E**) Males, (**F**) Females. “Re_abu” denotes the relative abundance of SARS-CoV-2 in PM_2.5_ samples, and “virus_log” denotes the log-transformed concentration of SARS-CoV-2 in wastewater samples. The dashed line represents the null line (regression coefficient β = 0). An effect is considered statistically significant when the 95% confidence interval does not cross this line.

The lag relationships between SARS-CoV-2 levels in PM_2.5_ and wastewater and the predicted COVID-19 case counts are presented in Figure 10. The viral concentration in wastewater had the strongest effect on disease incidence at lag 0 days, leading to an increase of 238.17–550.40 cases in the total population, with the ≥65-year group exhibiting the largest risk increment. For airborne transmission, the PM_2.5_-associated viral load showed the most pronounced effect at a 2-day lag, resulting in an increase of 507.47–2531.08 predicted cases across the total population, with the elderly group being most affected. Gender-stratified analyses (Figure 10E–F) revealed distinct patterns between the two media. For wastewater-borne SARS-CoV-2, effect estimates were consistently higher in females than in males across all lag periods, with the strongest effect at lag 0 days, where each one-unit increase in log viral concentration corresponded to an increase of 102.81 to 227.54 predicted cases in males and 131.13 to 347.85 in females. For PM_2.5_-associated SARS-CoV-2, effect estimates were consistently higher in males than in females across all lag periods, peaking at lag 2 days, where each unit increase in viral concentration corresponded to an increase of 246.28 to 1058.07 predicted cases in males and 196.46 to 1598.15 in females. These findings suggest that wastewater-borne viruses may have a greater impact on women, while airborne viruses may pose a higher risk to men, providing an important basis for formulating precise prevention and control strategies for different populations.

## 4. Discussion

This study found that the level of SARS-CoV-2 carried by PM_2.5_ was positively correlated with CO, PM_10_, NO_2_, and atmospheric pressure, but negatively correlated with temperature and sunshine duration. These findings are generally consistent with previous studies [12,13]. Existing evidence suggests that higher CO concentrations may be associated with a higher risk of COVID-19 incidence [14], while air pollutants such as PM_2.5_, PM_10_, and NO_2_ have also been considered potentially related to epidemic transmission [15,16]. In this study, the positive association between atmospheric pressure and viral levels may be attributed to stable atmospheric stratification under high-pressure conditions, which restricts pollutant dispersion and promotes the local accumulation of virus-containing particles [17,18]. In contrast, higher temperatures and longer sunshine duration may reduce viral persistence in the environment by enhancing air circulation and ultraviolet-mediated viral inactivation [19], thereby exerting a negative effect on concentrations in PM_2.5_. However, the monitoring period of this study was relatively limited, and continuous surveillance is needed to further reveal the spatiotemporal patterns of viral aerosol transmission.

SARS-CoV-2 concentrations in wastewater were negatively correlated with SO_2_, which is consistent with previous findings and suggests that SO_2_ may have an indirect inhibitory effect on the virus [20]. In addition, precipitation and humidity were positively associated with viral concentrations, indicating that humid environments may favor viral stability and persistence in aquatic systems [21,22]. Experimental evidence has shown that the median half-life of SARS-CoV-2 exceeds 24 h at 10 °C and 40% relative humidity, but decreases to only 1.5 h at 27 °C and 65% relative humidity [23]. This suggests that low-temperature and low-humidity conditions may facilitate viral survival. Therefore, under favorable climatic conditions, wastewater systems may serve as important media for the persistent presence of viral signals, highlighting the need to strengthen seasonal monitoring and treatment measures.

Current evidence regarding sex differences in COVID-19 incidence remains inconsistent. Some studies have reported a higher incidence among males, whereas others found no significant difference or even a higher incidence among females [24,25,26]. These discrepancies may be attributed to differences in study period, region, and population structure. In this study, the 22–49-year age group accounted for the highest proportion of confirmed cases, which is consistent with previous findings and may reflect more frequent social activities and greater exposure opportunities in this population [27].

The association between environmental factors and COVID-19 incidence showed a negative correlation between CO and case numbers. At lag 0 and lag 14 days, the relationship between CO concentration and COVID-19 risk exhibited a nonlinear increase-then-decrease pattern, suggesting a short-term threshold effect. The identified threshold concentration of 0.5 mg/m^3^ may provide a reference for regional air quality management. Although most studies have reported a positive association between CO concentration and COVID-19 incidence [28], the negative trend observed in this study may be related to the generally good air quality and relatively low CO levels in Fuzhou, indicating that the effect of air pollution on viral transmission may be dose-dependent.

In the GAM, SO_2_ concentrations above 5.0 μg/m^3^ were associated with reduced risk, whereas the DLNM showed that standardized changes in SO_2_ concentration were associated with increased risk, reflecting potential differences between short-term exposure and long-term lagged effects. In addition, O_3_-8 h was associated with reduced risk at 60–80 μg/m^3^, while no significant effect was observed at higher concentrations. This may be related to the dual role of ozone, which can inactivate viruses through strong oxidation but may also induce inflammatory responses in hosts [29]. Further validation through viral survival experiments is needed. Overall, this study reveals the complex associations between COVID-19 transmission and environmental factors in Fuzhou, emphasizing the importance of multi-model integration and spatiotemporal heterogeneity analysis. These findings provide scientific evidence for epidemic prevention and control, although long-term follow-up and interdisciplinary collaboration are still needed to strengthen the evidence chain.

Distinct lag effects were observed between different environmental media. The effect of PM_2.5_-carried SARS-CoV-2 was strongest at a lag of 2 days, while wastewater viral concentrations showed the strongest effect at lag 0. However, it should be noted that the predicted COVID-19 cases used in this analysis were derived from sentinel hospital surveillance, which inherently involves delays related to symptom onset, healthcare-seeking behavior, and laboratory confirmation. Therefore, the observed lag effects likely represent upper-bound estimates of the true biological lags. Nevertheless, this difference may still be related to viral transmission pathways and to differences in persistence characteristics across different media. Previous studies have shown that airborne fine particles can serve as adsorption carriers for viruses, promoting longer suspension and long-distance transport in aerosols [30]. As an aerosol carrier, PM_2.5_ may show a more evident lag effect due to atmospheric dispersion and deposition processes, whereas wastewater may more directly reflect population-level viral shedding [31,32]. Based on these differences, wastewater monitoring may be more suitable for capturing early epidemic signals, whereas PM_2.5_ monitoring may be more useful for assessing short-term transmission risk. Their combined application may provide a more comprehensive understanding of epidemic dynamics.

Age- and sex-stratified analyses further revealed heterogeneity in viral exposure. PM_2.5_-carried SARS-CoV-2 showed no significant effect in the 0–14-year age group, whereas the predicted number of cases increased markedly at a 2-day lag in the 15–64-year and ≥65-year groups, possibly due to reduced immune function and differences in outdoor exposure patterns among older adults [33]. Females showed a broader response range to wastewater viral signals, while males exhibited a stronger response to PM_2.5_-carried viral signals. These differences may be related to occupational exposure patterns and physiological characteristics. These findings suggest that multimedia surveillance can improve the sensitivity of epidemic early warning and provide support for targeted prevention and control strategies in specific populations.

## Figures and Tables

**Table 1:** Basic characteristics of air quality and meteorological factors in Fuzhou, 2023.

Variable	Mean ± SD	Minimum	Median	Maximum	Interquartile Range
PM_10_ (μg/m^3^)	37.64 ± 21.25	7	34	241	21
PM_2.5_ (μg/m^3^)	20.44 ± 12.36	2	18	91	14
NO_2_ (μg/m^3^)	16 ± 7.01	2	15	39	9
SO_2_ (μg/m^3^)	3.92 ± 0.80	2	4	7	1
CO (mg/m^3^)	0.47 ± 0.11	0.28	0.45	0.90	0.12
O_3_-8 (μg/m^3^)	110.04 ± 32.10	13	110	194	47
Atmospheric Pressure (hPa)	997 ± 39.67	768	1002	1025	12
Temperature (°C)	21.68 ± 7.38	5.20	22.30	33.70	13.40
Relative Humidity (%)	73.97 ± 12.12	42	72	100	19
Sunshine Duration (h/d)	4.93 ± 3.99	0	4.80	12.50	8.10
Wind Speed (m/s)	1.94 ± 0.64	0.70	1.80	4.50	0.80
Minimum Visibility (m)	8931.06 ± 7588.74	132	7250	30,000	10,411
Precipitation (mm)	6.02 ± 22.36	0	0	295.60	2.60

**Table 2:** Stepwise regression analysis of air quality and meteorological factors on SARS-CoV-2 levels.

Variable	β	t	*p*
CO	0.290	2.380	0.018 *
NO_2_	0.003	1.494	0.136
O_3_-8	−0.001	−1.875	0.062
PM_2.5_	0.002	1.937	0.054
PM_10_	0.001	1.580	0.115
SO_2_	−0.006	−0.367	0.714
Atmospheric pressure	0.001	2.051	0.041 *
Temperature	−0.009	−5.097	<0.01 **
Relative humidity	−9.64 × 10^−5^	−0.087	0.931
Precipitation	−0.001	−1.245	0.214
Daily mean wind speed	−0.040	−1.945	0.053
Sunshine duration	−0.012	−3.545	<0.01 **
Minimum visibility	−2.84 × 10^−6^	−1.607	0.109

Note: * *p* < 0.05, ** *p* < 0.01.

**Table 3:** Stepwise regression analysis of air quality and meteorological factors on SARS-CoV-2 levels in wastewater.

Variable	β	t	*p*
CO	−0.008	−0.059	0.953
NO_2_	−0.148	−1.087	0.282
O_3_-8	0.095	0.717	0.477
PM_2.5_	−0.089	−0.658	0.513
PM_10_	−0.215	−1.591	0.118
SO_2_	−1.432	−2.886	0.006 **
Average atmospheric pressure	−0.037	−0.271	0.788
Average temperature	0.068	0.512	0.611
Average relative humidity	0.242	1.610	0.114
Precipitation	−0.038	−0.258	0.797
Daily mean wind speed	−0.125	−0.940	0.352
Sunshine duration	−0.006	−0.647	0.518
Minimum visibility	−0.051	0.381	0.705

Note: ** *p* < 0.01. Sunshine duration was excluded from this stepwise regression model due to non-significant association with wastewater SARS-CoV-2 concentrations.

**Table 4:** Age distribution of COVID-19 cases in Fuzhou, 2023.

Age	Number of Tested	Number of Positive	Positive Rate (%)
0~	505 (11.46%)	37 (8.10%)	7.33
3~	515 (11.69%)	31 (6.78%)	6.02
6~	1190 (27.01%)	160 (35.01%)	13.45
22~	1130 (25.65%)	153 (33.48%)	13.54
49~	396 (8.99%)	40(8.75%)	10.10
65~	669 (15.19%)	36 (7.88%)	5.38
Total	4405	457	10.37

## Data Availability

The data analyzed in this study is subject to the following licenses/restrictions: the data are not publicly available due to privacy. Requests to access these datasets should be directed to wcc@fjmu.edu.cn.

## Abbreviations

The following abbreviations are used in this manuscript:

PM_2.5_	Fine particulate matter
SARS-CoV-2	Severe acute respiratory syndrome coronavirus 2
COVID-19	Coronavirus disease 2019
RT-qPCR	Reverse transcription quantitative polymerase chain reaction
GAMs	Generalized additive models
DLNMs	Distributed lag nonlinear models
